# Biobased Castor Oil-Based Polyurethane Foams Grafted with Octadecylsilane-Modified Diatomite for Use as Eco-Friendly and Low-Cost Sorbents for Crude Oil Clean-Up Applications

**DOI:** 10.3390/polym14235310

**Published:** 2022-12-05

**Authors:** Helanka J. Perera, Anjali Goyal, Saeed M. Alhassan, Hussain Banu

**Affiliations:** 1 Maths and Natural Science, Abu Dhabi Women’s Campus, Higher Colleges of Technology, Abu Dhabi P.O. Box 25026, United Arab Emirates; 2 Department of Chemical Engineering, Khalifa University, Abu Dhabi P.O. Box 127788, United Arab Emirates

**Keywords:** castor oil, octadecyltrichlorosilane, sorbent, polyurethane, hydrophobicity

## Abstract

Herein we report the synthesis and characterization of novel castor oil-based polyurethane (PU) foam functionalized with octadecyltrichlorosilane (C18)-modified diatomaceous earth (DE) particles, exhibiting superior hydrophobicity and oil adsorption, and poor water absorption, for use in effective clean-up of crude oil spillage in water bodies. High-performance and low-cost sorbents have a tremendous attraction in oil spill clean-up applications. Recent studies have focused on the use of castor oil as a significant polyol that can be used as a biodegradable and eco-friendly raw material for the synthesis of PU. However, biobased in-house synthesis of foam modified with C18-DE particles has not yet been reported. This study involves the synthesis of PU using castor oil, further modification of castor oil-based PU using C18 silane, characterization studies and elucidation of oil adsorption capacity. The FTIR analysis confirmed the fusion of C18 silane particles inside the PU skeleton by adding the new functional group, and the XRD study signified the inclusion of crystalline peaks in amorphous pristine PU foam owing to the silane cross-link structure. Thermogravimetric analysis indicated improvement in thermal stability and high residual content after chemical modification with alkyl chain moieties. The SEM and EDX analyses showed the surface’s roughness and the incorporation of inorganic and organic elements into pristine PU foam. The contact angle analysis showed increased hydrophobicity of the modified PU foams treated with C18-DE particles. The oil absorption studies showed that the C18-DE-modified PU foam, in comparison with the unmodified one, exhibited a 2.91-fold increase in the oil adsorption capacity and a 3.44-fold decrease in the water absorbing nature. From these studies, it is understood that this novel foam can be considered as a potential candidate for cleaning up oil spillage on water bodies.

## 1. Introduction

Global theoretical data collected by researchers show that Middle Eastern countries occupy the top of a list of the most important sources of oil production, with an estimated contribution of about 28% of world oil exports [1]. The United Arab Emirates (UAE), a gulf country, produces about four to five million barrels per day, making it the seventh-largest producer in record lists [2]. However, about 20,000 oil tankers pass through the 70–80 nautical miles that stretch along the country’s East Coast each year. These tankers carry about 40 percent of the world’s oil [3]. The coastlines of gulf countries suffer from massive oil spills caused by illegal oil discharges, due to the breaking of storage tanks/pipelines, oil transportation, cleaning of tanks or transferring the oil to vessels. These threaten marine life, damage coral reefs, pollute beaches and adversely affect aquatic ecosystems. The crude oil sludge washes ashore, destroying boats, engines, and fishing gear, and polluting fisheries; dead and injured wildlife also disrupts salinity [4,5,6].

In recent decades, oil/water separation has become a focus of attraction for scientists who have developed methods to combat oil spills on open lands, including in situ burning, chemicals, bioremediation, and mechanical recovery to clean up oil spills and reduce pollution of water bodies [6,7]. The above-mentioned remediation methods have been used for years. Still, they are inefficient in terms of both labor and equipment, whereas sorption [8], which is classified as mechanical recovery, is considered the most practical and well-known approach for removing oil spills and preventing the spread of oil. This technique follows the combined mechanism of adsorption, absorption and desorption in cleaning oil spills. The oil from the polluted oil–water interlayer is absorbed by the sorption material anchored in the open structure of the material and later squeezed out by desorption for further recycling [9,10,11].

Polymeric adsorbents with porous morphology have become a fascinating field for oil cleansing in recent days because they have good hydrophobicity and oleophilicity, high specific surface area, and easy operation procedure, and are very inexpensive compared to other adsorbents. Various sorbent materials have been used in oil sorption tests, including organic mineral as cellulose fiber [12,13], polystyrene (PS) [14,15], polypropylene (PP) [16,17] and polyurethane (PU) [18,19,20] foams, which various research groups have investigated. The lightweight and open-cell PU foam gained popularity among other polymeric adsorbents due to high porosity, good elasticity, easy industrial production and high oil sorption at low and high viscosity of oils and derivatives [21,22,23]. However, the presence of PU moieties in the network reduces hydrophobicity, which can be improved by various techniques such as chemical vapor deposition [24], dip coating [25], in situ chemical reaction [26] and introduction of micro-nano particles [27]. In recent work, a one-step ultrasonic dip-coating process [28] was used to prepare a superhydrophobic sepiolite (SEP)-coated PU sponge for oil spill clean-up from the water’s surface. The PU foam was modified by superhydrophobic SEP powder functionalized with octadecyltrimethylammonium bromide (OTAB) and octadecyltrichlorosilane (OTS) to achieve high adsorption capacity, selectivity and separation performance as an adsorbent sponge. In addition, a superhydrophobic PU sponge adsorbent [29] was developed by mussel-inspired chemistry and in situ polymerization with 1-hexadecane, which exhibits exceptional mechanical properties and excellent adsorption of oils and organic solvents. A facile approach was disclosed to fabricate reduced graphene oxide-coated PU sponges [30] that exhibit high sorption capacity for oil and improved recyclability. Moreover, our recent publication exhibited improvement in hydrophobicity of PU foam by incorporating fluorosilane-modified diatomaceous particles [31].

The physical surface modification of commercial PU foam by nanoparticles sometimes becomes challenging due to nonuniform distribution or entrapment of particles over or inside the sponges, adversely affecting oil sorption capacity from the oil/water mixture. Therefore, more research has been emphasized on in situ PU formulations incorporating modified micro-nano particles during PU foam production. The detailed literature study about PU shows that the evaluation of the foam is accompanied by chemical modification with organosilane (triethoxyvinylsilane and tetraethoxysilane) [32], nanoclays [33], porous fibers [34] and biomass carbon [35] to improve roughness on the sponge surface by increasing the hydrophobicity and sorption capacity of oils. The surface structure of PU foam modified by montmorillonite [36] or cloisite 20A [33] nanoclay and lignin [37] used as a filler for removal of crude oil from contaminated water enhances the separation efficiency of oil, whereas PU foam that is chemically synthesized exhibits better oil sorption capacity. The lignin-based PU foam [38], which has also been functionalized with polydopamine reduced graphene oxide is a superhydrophobic absorbent for oil spill clean-up applications. Moreover, abundantly available, microporous and nontoxic natural filler material, diatomaceous earth (DE) [39], can be used to modify the PU foam surface. The appreciable characteristic of DE is its amazing amorphous silica micro-skeleton and biomineralization concept. The use of these exoskeletons of diatoms to coat smooth surfaces creates a hierarchical structure, resulting in an improved wettability property [40]. However, chemical modification of the DE substrate, which is modified with silane before use, improves hydrophobicity.

The PU industries primarily depend on depleting petroleum-based resources for synthesizing primary raw materials, such as polyols and isocyanates. To overcome this limitation, current researchers have focused on using sustainable resources from plant oils [26], starch [41], chitosan [42] and cellulose [43], intending to replace petroleum products. These substituent natural resources have innumerable advantages, such as being inexpensive, readily available and eco-friendly for synthesizing biodegradable PU foams to avoid pollution and waste disposal. Various vegetable oils, such as palm oil [44], sunflower oil [45], rapeseed oil [46], castor oil [47] and canola oils [48] can be used to synthesize PU foams. The triglycerides of castor oil, rich in oleic acid (90%) with a combination of hydroxyl and unsaturated multi-functionality in the fatty acid chains, can react with isocyanates to form urethane linkages, i.e., interpenetrating polymer networks in PU foams [49,50,51]. The use of castor oil for oil adsorption application has been reported by modifying the surface with liquefied lignin-based PU foams. A study by Alves [50] et al. reported a method for preparing castor oil-based PU foams with different polyol: isocyanate concentrations for diesel oil sorption. The use of açaí seeds [52] as an ecosorbent filler in castor oil-based PU and its applications in oil/water separators in grease traps in households and restaurants was demonstrated.

The current research emphasizes the synthesis of a sorbent used for crude oil removal based on an effective PU foam. The synthesis is based on the biobased polyol, i.e., castor oil, isocyanate, and surface modifying agent octadecyltrichlorosilane (C18) functionalized DE to separate a crude oil/water mixture effectively. Here, C18 can be anchored to the DE surface by covalent bonds between hydrolyzed silane and the hydroxyl group of silica on DE. These alkyl chains in C18 confer a good affinity for oil and minimize the surface free energy, facilitating improved oil absorption [53]. This study reports on the synthesis and characterization of flexible and high porous PU foam with the integration of long-chain C18 functionalized DE particles as surface modifying agents incorporated in situ during PU foam formulation. Modified and unmodified PU foams were characterized by Fourier transform infrared spectroscopy (FTIR), scanning electron microscopy (SEM), energy dispersive X-ray analysis (EDX), X-ray diffraction studies and thermogravimetric analysis (TGA) to investigate the changes in structural, morphological and thermal behaviors. The hydrophobicity and oil/water adsorption capacities were studied to investigate the PU foam’s performance further.

## 2. Experimental

### 2.1. Materials

Octadecyltrichlorosilane (C18) and castor oil were obtained from Sigma Aldrich (St. Louis, MO, USA). The crude oil was obtained from Abu Dhabi National Company (ADNOC) (Abu Dhabi, United Arab Emirates) and methylene diphenyl diisocyanate (MDI) was purchased from Dow Chemicals (VORANATE™ M 229 Polymeric MDI, Midland, MI, USA). Unmodified DE, hexane and pentane were obtained from Eastchem (Xinbei District, Changzhou, China), Pharmco-Aaper (Brookfield, CT, USA) and Merck (Kenilworth, NJ, USA), respectively. Deionized water (DI) was used throughout the experiments. All chemicals were used as received without further purification.

### 2.2. Preparation of C18-DE Modified Particles

In a 50 mL glass vial, hexane (25 mL), unmodified DE (1 g) and C18 (0.2 g) were added together. The reaction mixture was allowed to heat for 5 h at 50 °C on a magnetic stirrer at 2000 rpm. The solid particles were separated from the solution by centrifugation. Again, 50 mL hexane was used to wash unreacted C18 from the modified DE particles thrice to get the purified C18-DE product, followed by air drying in the oven for 2 days at 70 °C.

### 2.3. Synthesis of Polyurethane Foam (PU Foam)

PU foam without modification was synthesized for comparison with modified PU foam.

The PU foam was synthesized according to the formulation reported in Table 1 without C18-DE particles. The castor oil as polyol and blowing agents DI water and pentane were mixed in a small beaker at ambient temperature for 5 min; then, MDI was added and stirred for 30 s. Afterward, the resultant foaming mixture was immediately transferred into a test tube, where it was allowed to rise freely. At last, the foam was allowed to cure at ambient temperature for a minimum of 2 days.

### 2.4. Synthesis of PU-C18-DE Foam (Modified)

PU-C18-DE foam was synthesized as per the Table 1 formulation described and synthesis was carried out similarly, but the mere difference shown is the incorporation of 10% of C18-DE-modified particles in the mixture of castor oil and blowing agents before the addition of MDI. Then, after the addition of MDI, the mixture was stirred and transferred into a test tube, where it would rise and then finally be placed at room temperature for curing for at least 2 days.

## 3. Measurements and Characterization:

### 3.1. Fourier Transform Infrared (FTIR) Spectroscopy

FTIR spectra were obtained using a Perkin Elmer Frontier FTIR spectrometer (PerkinElmer Genetics Inc., Waltham, MA, USA). The scanning frequencies were from 400 to 4000 cm^−1^ with a spectral resolution of 4 cm^−1^ and 32 scans.

### 3.2. Measurement of Thermal Properties

The thermogravimetric analysis (TGA) was performed to compare the thermal stability of the modified PU foam with the unmodified PU, and to determine the C18-DE grafting in PU foam using a TA Instruments, model SDT 650 thermogravimetric analyzer (TA Instruments, New Castle, DE, USA). The samples were heated from 25 to 800 °C with a heating rate of 20 °C/min under 40 mL/min of inert nitrogen gas.

### 3.3. Morphology and Elemental Composition

The surface morphology and elemental composition were performed by scanning electron microscopy and energy dispersive X-ray analysis, using a FEG Quanta 250 (FEI Company, Hillsboro, OR, USA) instrument, of the unmodified and modified PU under high vacuum mode operated at an acceleration voltage of 5 kV and at a working distance of about 10 mm. For SEM studies, each sample was attached to a double-sided carbon adhesive tape on top of an aluminum stud. The samples were then made conductive by the sputtering of Au/Pd. The PU surfaces were analyzed using energy-dispersive X-ray spectroscopy (SEM-EDX) to determine the atomic percentage of present elements.

### 3.4. Crystallinity Measurement

Diffraction (XRD) patterns were collected using X’Pert PRO powder diffractometer (Cu-Kα radiation 1.5406 Å, 45 kV, 40 mA) in the range of 5–80°, 2θ scale.

### 3.5. Hydrophobicity

For contact angle measurements, the unmodified PU, DE, C18-DE and PU-C18-DE samples were attached to a glass slide by placing a piece of the sample with the help of double-sided stick tape. Water contact angle measurements were performed using the static drop method at room temperature using KRÜSS DSA25 Series (KRÜSS Scientific Instruments, Inc., Matthews, NC, USA). Deionized water was used as a probe liquid (0.3 µL dispense volume) at a frequency of 20 in a time interval of 3000 milliseconds. The water contact angle was calculated by taking the average of at least ten consecutive measurements.

### 3.6. Determination of Oil Sorption Capacity

To measure the oil and water sorption capacity of the unmodified and modified PU, the glass container was filled with crude oil (50 mL) or distilled water (50 mL) alone. Then, ~1 g of adsorbent material cubes of unmodified and modified PU foam was added to the crude oil or distilled water. Later, the system was left undisturbed for a period of 2, 4 and 20 min. Later, the foam was removed and weighed accurately to calculate the total mass of adsorbed oil or absorbed water from both the modified and unmodified PU foam. Then, the crude oil adsorption capacity and the distilled water absorption capacity were calculated using the formula:

(1) $$\frac{\left(m^{f}-m^{i}\right)}{m^{i}}$$

where, *m^f^* and *m^i^* represent the masses of the polymer foam before and after immersion in oil/distilled water, respectively. The experimental setup was repeated by adding about 2 g of crude oil into a glass beaker containing 50 mL of distilled water, followed by adding the modified or unmodified PU foam. Then, the system was left undisturbed for 2, 4 and 20 min, followed by removing the sponge from the crude oil–water mixture. Later, the adsorbent material was removed, dried overnight at room temperature to dry the water out, and then weighed accurately to calculate the total mass of adsorbed oil in both the modified and unmodified PU foam.

## 4. Results and Discussion

The reported work focused on developing novel material within two steps by modifying the silane treatment of cost-effective DE powders with C18, which has been successfully entrapped into the synthesis of an in situ PU sponge by using bioresource castor oil as polyol agent along with isocyanate raw material. In recent decades, biobased vegetable oils have attracted much attention from researchers and industries due to their low cost, nontoxic nature, biocompatibility, unique backbone chemistry and easy availability. The biobased vegetable oils are easier to select as polyol material because hydroxyl functionality can react with isocyanate to form PU foam. One of our earlier works discusses the effect of alkyltrimethoxysilanes of different lengths on the surface properties of silane-modified diatomaceous earth (DE) [53].

The main objective of using the DE is to overcome the limitation of the PU, which has an inherently hydrophilic nature, and to impart the hydrophobic nature; hydrophobic chemical groups are decorated on the surface of PU. Earlier studies have used hydrophobic agents such as sepiolite and graphene oxide to improve hydrophobicity. In our earlier studies, we have used fluorosilane-modified diatomaceous earth particles and C18 silane that provides superhydrophobicity to the polyurethane, due to the fact that silica-based materials have hydrophobic moieties attached to their surfaces. Besides their inherent siliceous chemical nature, the coated DE particles, on the smooth surface skeleton of PU, are of a size 20 to 40 µm with innumerous micro or nano pores, as noted in the SEM studies. The enhanced surface roughness of the DE-coated PU very much enhances the hydrophobicity of the polymer matrix. Apart from the property of enhancing hydrophobicity, the DE particles also act as nucleating agents favoring the surface conjugation of chemical groups. The silane coupling agent immobilized onto the silica surface through the covalent bond greatly improves the surface hydrophobicity of the polymer matrix. The following characterization studies help us understand the surface microstructure and the successful biofunctionalization of the polymer to improve the hydrophobicity and high sorption capacity.

### 4.1. X-ray Diffraction Analysis of Synthesized Samples

**Figure 1:**
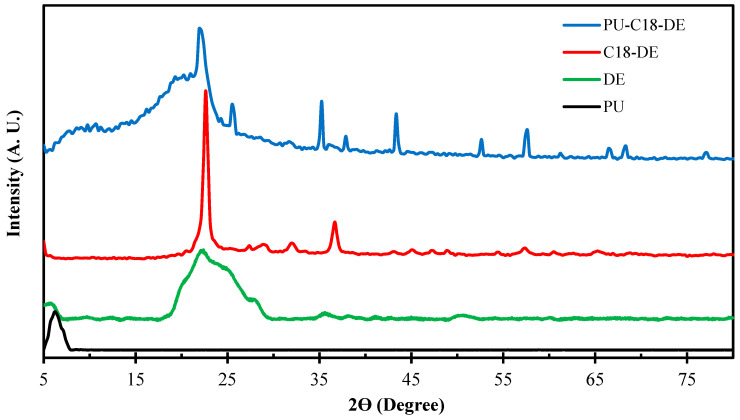
Powder XRD spectra of PU (bottom), DE, C18-DE particles and PU-C18-DE (top). XRD spectra of PU-C18-DE show an increment in crystallinity due to the incorporation of modified silane DE particles in amorphous PU foam.

X-ray diffraction studies were performed to analyze the crystallinity of the synthesized samples and determine the dispersion state of modified C18-DE particles in PU foam. The results of XRD for all four samples, DE, C18-DE, pristine PU foam and PU-C18-DE foam, are shown in Figure 1.

The XRD spectra shown in Figure 1, the unmodified DE powder, consists of amorphous silica and a broad peak at 22°, which can be attributed to the crystalline quartz property of diatomite [54]. In contrast, some small peaks can be due to small impurities in the DE. On the contrary, synthesized pristine PU indicates an amorphous phase and no crystalline peak is observed all over the spectra. Moreover, DE particles modified with C18 alkyl silane spectra exhibit a sharp crystalline peak around 23°, confirming the intercalation of C18 on DE particles [55]. Moreover, C18-DE-modified particles added at the time of PU synthesis raised the crystallinity level of PU-C18-DE from the amorphous phase, which shows that the degree of crystallinity increased, as can be seen in the XRD spectra. However, it is important to note that when the dispersion of modified particles in the foam structure was carried out, the characteristic XRD peak of C18-DE particles shifted to lower angles owing to the penetration of the polymer chains into the interlayer space of the modified particles [33]. These results point out the successful loading of particles within the polymer matrix.

### 4.2. Scanning Electron Microscopy (SEM)

**Figure 2:**
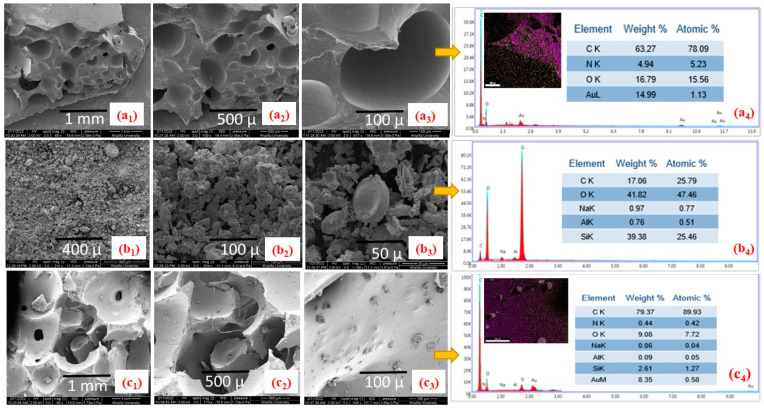
SEM images on different scales: (**a1**–**a3**) pristine PU synthesized foam, (**b1**–**b3**) C18-DE, (**c1**–**c3**) modified PU-C18-DE foam, EDX graph mapping and elemental analysis for all three samples, respectively, (**a4**), (**b4**), (**c4**).

SEM images of the PU foam with and without C18-DE particles, along with silane-modified DE particles, are shown in Figure 2. The SEM micrographs of PU foam without silane-modified DE particles displayed a smooth skeleton network of porous structure without any irregularities (Figure 2(a1–a3)). When DE particles are modified with C18 silane, as shown in Figure 2(b1–b3), they provide visual evidence of DE retaining disk-shaped regular and irregular particles. Moreover, incorporating C18 silane-modified DE particles during the formulation of PU foam developed a roughness on the surface that can be seen in Figure 2(c1–c3) [56]. This surface roughness is attributed to the presence of modified DE particles on the smooth skeleton of the PU foam matrix. Additionally, modified DE particles displayed homogenous dispersion on the PU surface. Our previous work on commercial PU foam treated with fluoro- and nonfluorosilane-modified DE particles controlled several microporous cells covered with silica fillers acting as nucleating agent [31,57]. In a similar manner, DE particles modified with fluoro-silanization deposited on a glass substrate improved the surface roughness with superhydrophobicity [39]. EDX and elemental analysis data clearly indicate the incorporation of C18-DE particles in the PU skeleton. In Figure 2(b4), modified C18-DE particles represent the Si, Al and Na as some of the constituents, which were absent in Figure 2(a4). In contrast, the modified DE, when engrossed in the PU matrix, exhibits the presence of all elements, which signifies the successful completion of the reaction [Figure 2(c4)].

### 4.3. Thermogravimetric Analysis

**Figure 3:**
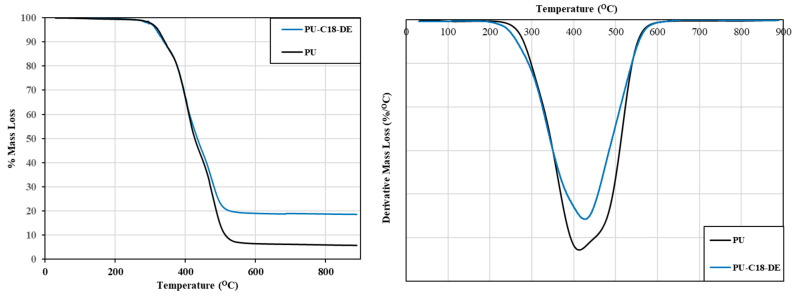
TGA (**left** image) and the corresponding DTA curve (**right** image) of PU and PU-C18-DE.

The thermograms shown in Figure 3 (left image) illustrate the thermal decomposition of unmodified castor oil-based PU with that of the castor oil-based PU after incorporating the silane coupling agent-modified DE particles under identical conditions. For both samples, the onset decomposition temperature started uniformly at around 250 °C, in accordance with the earlier TGA studies based on castor oil-based PU [47,58].

Further, both samples’ consequent mass loss events occurred in four major decomposition stages in multiple steps. The first degradation was observed between 250 to 300 °C and was mainly attributed to the degradation of urethane linkage of the castor oil. The second decomposition occurred between 300 to 350 °C due to the liberation of free isocyanate [58]. The third stage of degradation was between 350 to 550 °C, attributable to the degradation of a flexible segment of the hydroxyl moieties of castor oil [58,59,60]. According to the literature, C18-DE showed a broad mass loss from 250 to 800 °C, which is indicative of the breakdown of the alkyl chain moiety of the octadecylsilane [53]. The overlapping of the decomposition temperatures of pristine PU and PU-C18-DE can be clearly seen in Figure 3.

The thermograms’ mass loss % at 800 °C are 6.0% and 18.8% for the unmodified and modified PU, wherein the higher final mass loss % was observed in the case of modified PU foam arising from a remaining Si−O−Si moiety [55]. Due to the decomposition of C18 silane coupling agent, the mass loss of PU-C18-DE was higher at 18.8%, compared to unmodified PU. The difference of mass loss % at 800 °C was around 12.8%, and may be attributed to grafted C18-DE. In a similar study by Yuri Lvov et al., the residual mass % of polysiloxane-modified halloysite nanotube PU foam (POS@HNT-PUF) had higher residual mass % compared to uncoated PU foam [61]. The incorporation of silane did not significantly change the degradation; however, it increased the amount of char residue formed in PU-C18-DE, which was observed in Figure 3. Biobased PU prepared from modified castor oil and Kraft lignin also exhibited thermal stability and also had higher char residues after modification [62]. From the Differential Thermal Analysis (DTA) curve (Figure 3—right image), we understand that the observed mass losses are very close to each other and there is overlapping of the mass losses, resulting in one distinct peak; the individual mass losses are not very evident from the DTA curve.

### 4.4. Fourier Transform Infrared Spectroscopy (FTIR)

**Figure 4:**
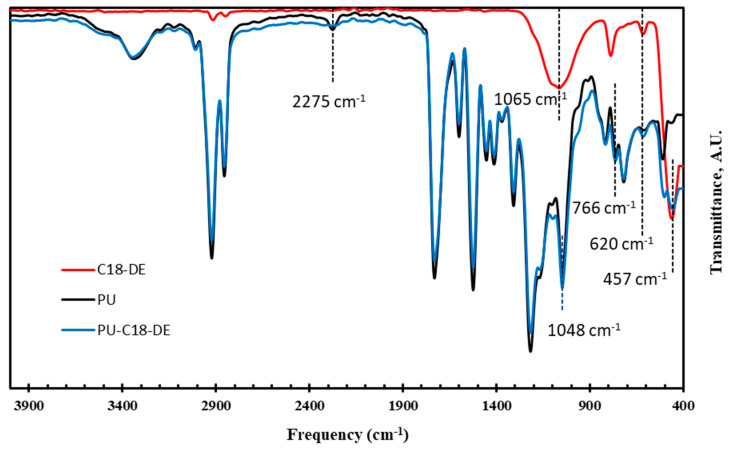
FTIR spectrum represents the structural changes of C18 grafted DE particles and synthesized PU foam before and after modification with C18-DE particles.

In order to confirm the C18 grafting on DE particles, the castor oil-based PU foam, with and without grafting C18-DE silane agents, was analyzed by FTIR analysis. The FTIR spectra of all samples, pristine PU foam, C18-DE particles and modified PU-C18-DE foam, are shown in Figure 4. The detailed explanation of peaks from the spectra is discussed in Table 2. The addition and removal of peaks based on the difference between functional groups in PU foam after and before modification has been clearly mentioned. In our previous work, FTIR spectra of neat DE has been discussed [31]. The current FTIR spectra depict the grafting of the C18 silane coupling agent on DE particles. The addition of FTIR peaks at 2911, 2847 cm^−1^, due to CH_2_ asymmetric and symmetric stretching in C18-DE samples, confirms the grafting of the C18 agent on the surface of DE particles, which was absent in pure DE sample spectra [53]. The FTIR spectrum of pristine PU foam and the PU-C18-DE confirmed the presence of urethane linkages, as represented by a vibrational region mainly at 3340 cm^−1^ due to -NH asymmetric stretching, and 2924 cm^−1^ and 2858 cm^−1^, attributed to C−H stretching of asymmetric and symmetric. The peaks at 1733, 1610 and 1522 cm^−1^ are associated with C=O stretching in amide, urea and NH deformation in the amide (II) band. The peak at 1050 cm^−1^ corresponds to ester C−O−C stretching vibration [63]. The incorporation of C18-DE particles in PU foam by FTIR spectra can be confirmed by the disappearance of the peak at 2275 cm^−1^ from the modified PU foam because it corresponds to free, unreacted isocyanate monomer in PU structure [56]. Other major new peaks appeared in modified PU foam at 766, 620 and 457 cm^−1^, attributed to Si−OH vibration/Si−CH_3_ polysiloxane, Si−O vibration of silica and Si−O−Si bending vibrations [64,65]. The availability of these peaks in the modified PU foam confirmed the entrapment of C18-DE modifiers. Moreover, the EDX results also favor the FTIR spectra that the chemical interactions between the C18 silane coupling agent and DE surface, along with modification in PU foam, have been successfully performed.

### 4.5. Surface Wettability Measurement

**Figure 5:**
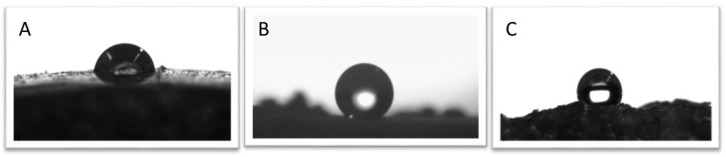
The water contact angle of (**A**) unmodified PU is 85.5°, (**B**) C18-DE is 137.2° (**C**) C18-DE-PU is 129.8°.

The images and graphical representation for the water contact angle of PU foam, C18-DE and PU-C18-DE foam are measured and shown in Figure 5. It is shown that after the modification with silane-modified DE particles, the surface of the PU foam has some irregularities, because silane-modified DE particles improve the hydrophobicity by developing the roughness on the surface and lowering the free energy that imparts the water-repellent nature. According to the literature, diatomite particles modified with the C18 silane coupling agent enhance the water contact angle. Enhancement of contact angle is attributed to the attachment of the alkyl group with hydroxyl in diatomite that lowers the surface energy level and increases roughness on the surface with the introduction of DE particles [39,53]. Herein, the water contact angle of pristine PU was improved when it was modified with long-chain C18 silane DE particles. The literature study reveals perpendicular alignment of long alkyl chains that include low surface energy, as alkyl chains (*n* ≥ 10) have a tendency to retrieve the surface from water because of low surface energy. These long chains protect the hydrophilic groups on the DE surfaces, as alkyl chains were grafted on these groups. Moreover, the inclusion of diatomite particles is also arranged in an ordered manner that enhances the chance to improve hydrophobicity on the surface of samples [39,53]. Figure 5 shows the water contact angle of unmodified PU and PU after grafting with C18-DE; it shows a shift in hydrophilicity from 85.5° to 129.8°. This contact angle increment was due to the low surface tension of silane coupling agents, which formed a cross-linked Si−O−Si network. This resists the water molecules entrapped into the PU skeleton [66], which was in good agreement with SEM images of the modified PU sample (Figure 2c1–c4). The results also show consistency with our previous latest work, which represents the physical entrapment of C18-DE on commercial PU sponges [57]. This modification favors the strong hydrophobicity of samples that can be used in oil absorption.

### 4.6. Determination of Oil Sorption and Water Absorption Capacity of Polymer

**Figure 6:**
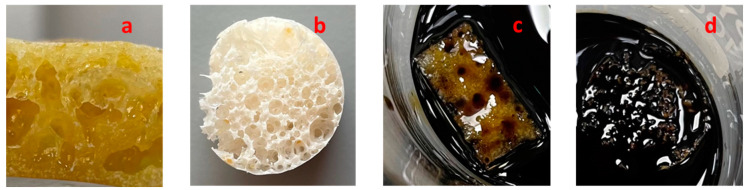
Represents the oil absorption studies in the biobased PU (**a**) unmodified PU, (**b**) PU-C18-DE, after in contact with crude oil for 20 min, (**c**) unmodified PU and (**d**) PU-C18-DE.

The oil adsorption of the castor oil-based PU foam with and without modification after immersion of foam in 50 mL of crude oil or 50 mL of distilled water is represented in Figure 6, wherein Figure 6a illustrates castor oil-based PU foam without modification and Figure 6b represents the castor oil-based PU foam modified with C18-DE(PU-C18-DE); with the increase in the time of contact of the modified foam with the crude oil (20 min), increased oil adsorption is very evident (Figure 6d) in comparison with the unmodified foam (Figure 6c).

The oil and water adsorption capacity for PU and PU-C18-DE are shown in Table 3 and Table 4, respectively. Compared to the unmodified PU foam, the PU-C18-DE foam exhibited a 2.91-fold increase in the oil-adsorption nature and a 3.44-fold decrease in the water-absorbing nature. The results showed that the PU-C18-DE foam exhibited superior oil adsorption and very poor water absorption, making the novel foam more suitable for absorbing crude oil. These results of the current study are in accordance with our previous research focusing on the surface conjugation of commercial PU with fluorosilane and DE; commercial PU coated with DE and C18 silane for use in crude oil clearance from water bodies [31,57].

**Figure 7:**
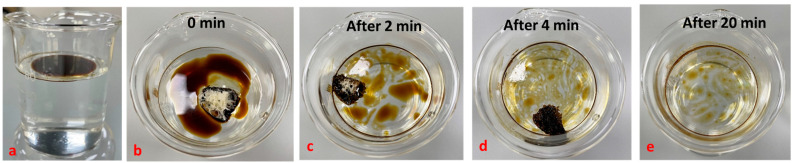
This figure illustrates the oil adsorption capacity of castor oil-based PU foam modified with DE-C18 studied by layering 1 g of crude oil (1–2 mm in thickness) on distilled water; the oil absorption at initial (**a**), 0 min (**b**), 2 min (**c**), 4 min (**d**), and after 20 min (**e**), respectively.

In a similar oil absorption study (Figure 7), 1 g of crude oil was layered (1–2 mm in thickness) on 80 mL of distilled water (Figure 7a), and the modified and the unmodified foams were immersed in the crude oil layered water for 0 (Figure 7b), 2 (Figure 7c), 4 (Figure 7d) and 20 min (Figure 7e), respectively. With the increase in the time of contact of the modified foam with the oil, there was increased oil adsorption noted and, especially after 20 min, most of the crude oil residues were cleared from the water surface. These results suggest that the modified PU foam can be used as a potential candidate for clearing crude oil from water bodies. Studies by Visco A. et al. on PU foams loaded with carbon nanofibers exhibited selective oil absorption properties in a water–oil mixture [67]. There are also other research works on surface modifications of polyurethane using various chemical moieties and nanomaterials for enhanced oil absorption reported [68,69,70,71,72,73]. There are several recent research works reported in the development of polymer foams for oil–water separations, which elucidates the potential of these foams as promising agents for removing oil from water bodies [74,75,76,77,78].

In our previous study involving commercial polyurethane foam surface modified with fluorosilane and diatomaceous earth, about a 1.67-fold high absorption of crude oil in comparison with the pristine commercial polyurethane foam has been noted. Similarly, polyurethane grafted with diatomaceous earth and C18 silane exhibited a 2.13-fold increase in oil absorption over commercial polyurethane foam. In the current research dealing with biobased polyurethane foam synthesized from castor oil surface functionalized with diatomaceous earth and C18 silane moieties, a superior oil adsorption of 2.91-fold increase is observed when compared to the pristine castor oil-based polyurethane. From the current studies, we understand that the biobased polyurethane with diatomaceous earth and silane exhibits a superior oil absorption capacity than that of pristine biobased polyurethane, as well as commercial polyurethane, in addition to the commercial polyurethane with diatomaceous earth and fluorosilane/C18 silane modifications [31,57].

In summary, this research mainly involves the development of a biodegradable polyurethane polymer synthesized using eco-friendly castor oil as the raw material and conferring superhydrophobicity to the less hydrophobic polyurethane by conjugating with diatomaceous earth that acts as a nucleating agent, as well as the substrate that increases the surface roughness, porosity and oil adsorption capacity. In addition to the above chemical decoration of the biobased polymer, C18 silane moieties are also coated to make it superhydrophobic with superior oleophilicity. The superhydrophobicity is investigated by studying the water contact angle and wettability; the ability to absorb oil and water is studied using the absorption capacity ratio of oil and water, thereby inferring the increased oleophilicity and decreased water absorption. The higher oil sorption capacity is mainly attributed to the porosity of the polyurethane polymer and the diatomaceous earth particle, and the poor water sorption is associated with the hydrophobic nature conferred by the surface roughness, due to the diatomaceous earth and C18 silane coating on the biobased polymer.

### 4.7. Conclusions

In summary, we have shown a straightforward synthetic route to prepare strong hydrophobic retained C18-DE particle-grafted PU foams and demonstrated the ability of the product for use as a sorbent for oil spill clean-up applications. The incorporation of DE-C18 lowered the surface energy, exhibited the cross-linking structure inside the PU skeleton and led to the corresponding improvement in macro and microscopic properties. The results of XRD, TGA, FTIR, SEM and contact angle measurements confirmed that alkylsilane with C18 chain-length DE particles were successfully grafted into the skeleton of hydrophilic PU foam. The FTIR studies revealed the fusion of C18 grafted DE particles into the PU foam noted by the appearance of some new peaks, which were absent in the pristine PU foam. The XRD studies showed that the rising crystalline peak in spectra of PU-C18-DE is due to silane moieties of C18-DE particles when compared to PU foam. The TGA study signified the better residual content of modified PU, owing to the availability of high alkyl chains converted into ash. The SEM images and the water contact angle measurement confirmed the roughness on a surface, which imparts hydrophobicity, and also the oil adsorption studies showed better oleophilicity and low water absorption, making the low-cost and eco-friendly sorbent material most suitable for enhanced oil recovery.

## Figures and Tables

**Table 1:** Formulation of chemical used for synthesis.

Chemicals Used	%
Polyol	63.7
Blowing agent: DI water, Pentane	0.6, 6.4
MDI	29.3
C18-DE(% by mass of all reactants)	10

**Table 2:** Significant peaks at FTIR spectra of pristine PU foam, C18-DE particles and PU-C18-DE foam.

Sample Code	Frequency (cm^−1^)			
	Vibration Bands	Expected Peaks	Observed Peaks	
Pristine PU foam	N-H stretching	3500–3200	3340	
	−CH stretching (Asy., Sy.) of the alkyl chain	2935–2847	2924, 2858	
	N=C=O stretching	2275–2240	2275	
	−(C=O) stretching of urethane and ester group	1745	1733	
	amide II: δN–H + νC–N + νC–C; sensitive to chain formation and intermolecular hydrogen bonding	1600–1500	1610	
	NH deformation (stretching vibrations)	1530–1500	1522	
	Isocyanurates	1420–1410	1417	
	C−O−C group	1250–1210	1217	
	C−O stretching	1070–1030	1050	
	CH_2_ rocking	730–710	717	
C18-DE	−CH stretching (Asy, Sy) of the alkyl chain	2935–2847	2911, 2847	
	Si−O, stretching	1120–1050	1065	
	Si−O−Si Sy. stretching	795–750	791	
	Si−O vib. of polymorphic silica	640–620	628	
	Siloxane bonds, Assy. stretching	480–450	465	
PU-C18-DE foam	N−H stretching	3500–3200	3352	
	−CH stretching (Asy., Sy.) of the alkyl chain	2935–2847	2924, 2856	
	N=C=O stretching	2275–2240	2275	Disappeared
	−(C=O) stretching of urethane and ester group	1745	1733	
	amide II: δN–H + νC–N+ νC–C; sensitive to chain formation and intermolecular hydrogen bonding	1600–1500	1610	
	NH deformation (stretching vibrations)	1530–1500	1522	
	Isocyanates	1420–1410	1417	
	C−O−C group	1250–1210	1217	
	C–O & Si−O−Si stretching	1070–1030	1048	
	Si−OH		766	Appeared
	CH_2_ rocking	730–710	721	
	Si−O−Si vib. of polymorphic silica		620	Appeared
	Siloxane bonds, Assy. stretching	480–450	457	Appeared

**Table 3:** Calculation of oil adsorption percentage to understand oleophilicity of the PU and PU-C18-DE.

Polymer Material	$m^{i}\;\mathrm{Value}$	$m^{f}\;\mathrm{Value}$	$\frac{\left(m^{f}-m^{i}\right)}{m^{i}}$	Average Oil Adsorption %
PU	1.02	1.49	0.452	45.2%
PU-C18-DE	1.13	2.86	1.73	131.6%

**Table 4:** Calculation of water absorption percentage to understand hydrophobicity of the unmodified and modified PU foam.

Polymer Material	$m^{i}\;\mathrm{Value}$	$m^{f}\;\mathrm{Value}$	$\frac{\left(m^{f}-m^{i}\right)}{m^{i}}$	Average Water Absorption %
PU	1.2	1.89	0.575	57.5%
PU-C18-DE	1.2	1.4	0.167	16.7%

## Data Availability

All data generated or analyzed during this study are included in this published article. The datasets used and/or analyzed during the current study are available from the corresponding author on reasonable request.
